# A 19-miRNA Support Vector Machine classifier and a 6-miRNA risk score system designed for ovarian cancer patients

**DOI:** 10.3892/or.2019.7385

**Published:** 2019-10-23

**Authors:** Jingwei Dong, Mingjun Xu

Oncol Rep 41: 3233-3243, 2019; DOI: 10.3892/or.2019.7108

Subsequently to the publication of this paper, the authors have realized that the affilitation address omitted the name of the university at which this research was conducted (Capital Medical University). Therefore, the author affiliations for this paper should have appeared as follows (changes are highlighted in bold):

JINGWEI DONG and MINGJUN XU

Department of Anesthesiology, Beijing Obstetrics and Gynecology Hospital, **Capital Medical University**, Beijing 100001, P.R. China

The authors regret that the name of the university was not included with the paper prior to its publication, and apologize to the readers for any inconvenience caused.

